# AM/PM dosing of LAMA for COPD: a randomized controlled trial protocol using digital recruitment and registries

**DOI:** 10.3389/fmed.2024.1430169

**Published:** 2024-08-06

**Authors:** Pradeesh Sivapalan, Valdemar Rømer, Tobias Wirenfeldt Klausen, Niklas Dyrby Johansen, Manan Pareek, Daniel Modin, Alexander Mathioudakis, Jørgen Vestbo, Josefin Eklöf, Alexander Jordan, John R. Hurst, Tor Biering-Sørensen, Jens-Ulrik Jensen

**Affiliations:** ^1^Department of Medicine, Respiratory Medicine Section, Copenhagen University Hospital-Herlev and Gentofte, Copenhagen, Denmark; ^2^Department of Clinical Medicine, Faculty of Health and Medical Sciences, University of Copenhagen, Copenhagen, Denmark; ^3^Department of Cardiology, Copenhagen University Hospital-Herlev and Gentofte, Copenhagen, Denmark; ^4^Division of Infection, Immunity and Respiratory Medicine, University of Manchester, Manchester, United Kingdom; ^5^North West Lung Centre, Wythenshawe Hospital, Manchester University NHS Foundation Trust, Manchester, United Kingdom; ^6^UCL Respiratory, University College London, London, United Kingdom; ^7^Center for Translational Cardiology and Pragmatic Randomized Trials, Department of Biomedical Sciences, Faculty of Health and Medical Sciences, University of Copenhagen, Hellerup, Denmark; ^8^Steno Diabetes Center Copenhagen, Herlev, Denmark

**Keywords:** long-acting muscarinic antagonists, chronic obstructive pulmonary disease, COPD exacerbation, all-cause mortality, intensive care admission

## Abstract

**Rationale:**

Long-acting muscarinic antagonists (LAMAs) reduce the risk of acute exacerbations of chronic obstructive pulmonary disease (AECOPD), usually taken once daily in the morning. However, the circadian activity of autonomic regulation suggests that the highest need for anticholinergic therapy may be in the late night/early morning. This is supported by evidence that AECOPD most often begins in the morning. Furthermore, the trough spirometry effect of LAMA is lower than the peak effect, which further argues that evening dosing may be more optimal than morning dosing. This trial aims to determine whether evening administration of LAMA reduces hospitalization-requiring AECOPD or death from all causes within 1 year as compared to morning administration of the same LAMA.

**Methods:**

Randomized controlled open-label trial. Persons aged 30 years or older with a once-daily LAMA prescription and a confirmed COPD diagnosis were recruited. Participants were randomized in a 1:1 ratio to either morning or evening LAMA administration. Complete follow-up for the primary outcome, hospitalization-requiring AECOPD, or death from all causes within 1 year was captured from the Danish National Health Register, as were patient-reported outcome assessments at 6 and 12 months.

**Results:**

A total of 10,013 participants were randomized, and the recruitment process started on 9 March 2023. Secondary outcomes include (i) moderate COPD exacerbations; (ii) all-cause hospitalization; (iii) ICU admission; (iv) need for non-invasive ventilation; and (v) all-cause mortality, among others. All outcomes will be evaluated 12 months after recruitment.

**Clinical trial registration:**ClinicalTrials.gov, NCT05563675.

## Introduction

### Background and rationale

One of the most serious complications of chronic obstructive pulmonary disease (COPD) is acute exacerbation (AECOPD). On average, each COPD patient experiences 0.5–3.5 acute exacerbations per year, which is an important reason for hospitalization, disease progression, and mortality as well as a decline in health status and lung function (1, 2). Treatment with a long-acting muscarinic antagonist (LAMA) reduces dyspnea and the risk of exacerbations in patients with COPD by binding to muscarinic receptors in bronchial smooth musculature and thus inhibiting cholinergic bronchial constriction. LAMAs are given as inhalation therapy once daily (most often) or twice daily (3). Most COPD patients experience their worst symptoms and experience exacerbations in the early morning hours, before getting out of bed (4). This might be explained by the physiological diurnal changes in the activity of the parasympathetic homeostasis system, which is most active at night to improve digestion and other secretions (5). Correspondingly, the activity of the sympathetic system is physiologically suppressed at night, and stimulation of β-2 receptors is thus also low (and opposite for M-3 receptors). The balance of sympathetic-parasympathetic tone is shifted significantly toward the latter. Most available LAMA treatments are dosed once daily in the morning. Thus, for a COPD patient, being at a trough level of LAMA (which antagonizes the parasympathetic system) at late night/early morning, may carry a hazard for the patient. Studies have found that lung function measured as a forced expiratory volume in 1 s (FEV1) improvement peaks approximately 2 h after LAMA administration and that FEV1 is still significantly improved at 7 h post-treatment but decreases toward the trough level of the LAMA (6). However, as a corollary to the above, when the medicine is probably most needed (02.00 a.m.–07.00 a.m.), the effect is at its lowest level, which may not be desirable, since a low effect of the most important preventive medicine against AECOPD at this time may lead to more exacerbations. Evening administration, on the contrary, would lead to a greater and more certain effect regarding bronchodilation and reduced secretion in the early morning hours, and a maximum effect should be expected during the entire night.

### Objectives

The main objective of this intervention and research protocol is to determine whether evening administration of LAMA, as opposed to morning administration, is superior for the occurrence of hospitalization-requiring AECOPD or death from all causes. The intervention will have the following secondary objectives: (1) moderate COPD exacerbations, (2) all-cause hospitalization, (3) intensive care unit (ICU) admission, (4) need for non-invasive ventilation, (5) all-cause mortality, (6) Short-Acting Beta-Agonist (SABA) use / pick up rate, and exploratory endpoints (1) change in COPD Assessment Test (CAT) score, (2) change in Medical Research Council (MRC) score, and (3) change in the “soundly sleeping” parameter of CAT.

### Trial design

The study is a pragmatic, registry-based, open-label, randomized controlled trial combining the utilization of the Danish nationwide health registries and the official Danish electronic letter system (Digital Post/e-Boks) into an innovative, decentralized trial requiring no study visits from participants. The trial will include patients receiving LAMA treatment that will be randomized 1:1 to either LAMA administration in the evening or the morning for 12 months.

## Methods: participants, interventions, and outcomes

### Study setting

This study will be conducted by the Chronic Obstructive Pulmonary Disease Trial Network (COP:TRIN) at the Section of Respiratory Medicine, Copenhagen University Hospital – Herlev and Gentofte, Copenhagen, Denmark. This group will be responsible for all processes in the study, including recruitment, inclusion, randomization, follow-up, and communication with participants. Registry data will be obtained through the Danish Health Data Authority and accessed through an encrypted remote-access server environment. The study will identify potential participants across Denmark through nationwide health registries, and the electronic letter system (Digital Post/e-Boks) will be used for sending recruitment letters and communicating with patients. The trial recruits and allocates patients electronically, and follow-up consists of electronic questionnaires, while endpoints are assessed using data from national registries. Therefore, there is no list of specific study sites/clinics/hospitals.

### Eligibility criteria

Inclusion criteria:

1. Age > 30 years.
2. Current treatment with LAMA once daily (as recorded in the Danish National Prescription Registry and confirmed by the participant via questionnaire).
3. Confirmed COPD diagnosis.

Exclusion criteria:

1. Patients who decline to participate.

### Who will obtain informed consent?

We have obtained a waiver from the regional science ethics board for informed consent since the medication and dose will not be changed. The science ethics board argues that the intervention lies beyond the scope of the science ethics legislation in Denmark.

However, only those who provided consent to the written information, and who reported having COPD, are included. We have obtained approval from the Data Protection Agency (P-2022-432) on 1 July 2022.

### Additional consent provisions for collection and use of participant data and biological specimens

No biological specimens will be obtained. Regarding informed consent, see previous section.

## Interventions

### Explanation for the choice of comparators

Patients will be randomized 1:1 to one of the two treatment strategies by computer-generated allocation to either:

- LAMA administration in the morning (control).
- LAMA administration in the evening (intervention).

These treatment strategies have been chosen because morning administration is the most conventional, while evening administration will be investigated to determine if it is superior with respect to the occurrence of hospitalization-requiring AECOPD or death from all causes within 12 months, along with several secondary and exploratory outcomes, as outlined in Table 1.

**Table 1:** Primary endpoint definition.

Endpoint	ICD-10 codes	Inpatient/outpatient/minimum required length of stay/other criteria
Hospitalization-requiring AECOPD or death from all causes	Hospitalizations: DJ440, DJ441	For hospitalizations: Type A Diagnosis (primary discharge diagnosis). Inpatient and length of stay ≥24 h, or emergency room visits

The time frame is ≥1 day after randomization until the end of the study (12 months). COPD, chronic obstructive pulmonary disease.

### Intervention description

Participants randomized to LAMA administration [with or without the combination of inhaled corticosteroids (ICSs) and/or long-acting beta-2-agonists (LABAs)] in the evening will be instructed to take their LAMA-containing inhalation between 8 p.m. and 2 a.m.

Participants randomized to LAMA administration (with or without the combination of ICS and/or LABA) in the morning will be instructed to take their LAMA as usual in the morning between 6 and 12 a.m.

### Criteria for discontinuing or modifying allocated interventions

Investigators may pause or interrupt the intervention at any given time in case of medical reasoning, safety risks, or requirements from the authorities.

**Figure 1:**
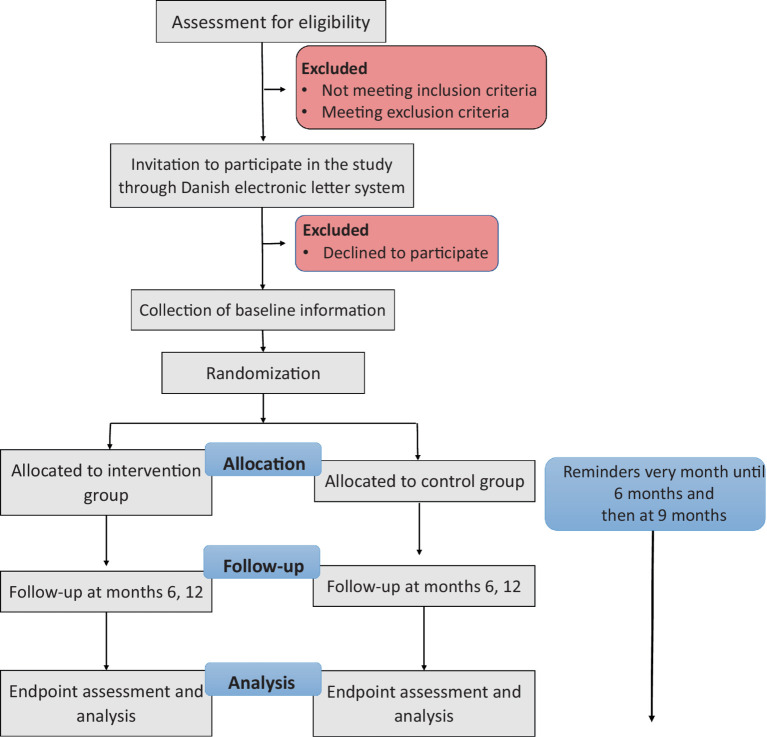
Clinical trial flowchart.

Furthermore, participating patients can withdraw from the intervention or the entire trial at any given time without providing a reason (Figure 1).

### Strategies to improve adherence to interventions

Reminders on LAMA administration time will be sent to participants every month until the first questionnaire (6 months), and then at 9 months.

### Relevant concomitant care permitted or prohibited during the trial

N/A: no specific concomitant care or interventions are prohibited during the trial.

### Provisions for post-trial care

The trial is covered by the patient compensation scheme if, contrary to expectation, damages occur during the trial and by inclusion.

### Outcomes

The primary efficacy endpoint is the occurrence of hospitalization requiring AECOPD or death from all causes within 12 months. Secondary efficacy endpoints included (i) moderate COPD exacerbations; (ii) all-cause hospitalization; (iii) ICU admission; (iv) need for non-invasive ventilation; (v) all-cause mortality; (vi) SABA use/pick-up rate; and exploratory endpoints (i) relation between adherence and AECOPD hospitalization, (ii) change in CAT score, (iii) change in MRC score, and (iv) change in “soundly sleeping” parameter of CAT.

The details on the primary and secondary outcomes of the study can be seen in Tables 1, 2, and exploratory outcomes in Table 3.

**Table 2:** Secondary and exploratory endpoint definition.

Endpoint	ICD-10/ATC codes	Inpatient/outpatient/minimum required length of stay/other criteria
Moderate COPD exacerbations	Prednisolone: H02AB06 or Antibiotics: J01CA04, J01AA02, J01CR02, J01MA14	Excluding hospitalizations and emergency room visits: DJ441 (A)
Hospitalization (all-cause)		Inpatient, length of stay ≥24 h
Hospitalization in ICU (all-cause)		Inpatient, ICU, length of stay ≥24 h
Need for NIV	BGDA1	A or B
Mortality (all-cause)		Participant registered as dead in the CPR register.
SABA use/pick-up rate	R03AC02, R03CC02, R03AC03	Danish National Prescription Registry
Change in CAT score	Questionnaire	At 6 and 12 months
Change in MRC score	Questionnaire	At 6 and 12 months

The time frame is ≥1 day after randomization until the end of the study (12 months from randomization) for all outcomes. A = primary discharge diagnosis and B = secondary discharge diagnosis. COPD, chronic obstructive pulmonary disease; ICU, intensive care unit; NIV, non-invasive ventilation; CPR, Central (National) Person Registry; SABA, short-acting beta-2 agonists; CAT, COPD assessment test.

**Table 3:** Exploratory endpoint definition.

Endpoint	Data source or ICD-10 codes	Inpatient/outpatient/minimum required length of stay/other criteria
Relation between adherence and hospitalization-requiring AECOPD	Questionnaire, DJ440, DJ441	For hospitalizations: Type A diagnosis (primary discharge diagnosis). Inpatient and length of stay ≥24 h, or emergency room visits. Deaths excluded.
Change in CAT score	Questionnaire	At 6 and 12 months
Change in MRC score	Questionnaire	At 6 and 12 months
Change in CAT “soundly sleeping” parameter	Questionnaire	At 6 and 12 months

A = primary discharge diagnosis. CAT, chronic obstructive pulmonary disease assessment test; MRC, Medical Research Council score.

### Participant timeline

The participant timeline is seen in Table 4.

**Table 4:** Participant timeline.

Event	Day 1	Months											
		1	2	3	4	5	6 months follow-up	7	8	9	10	11	12 months follow-up
Assessment for eligibility	*X*												
Acceptance of enrolment	*X*												
Randomization/allocation	*X*												
Questionnaire	*X*						*X*						*X*
Reminder of treatment allocation	*X*	*X*	*X*	*X*	*X*	*X*	*X*			*X*			*X*
Possibility to contact staff of trial	*X*	*X*	*X*	*X*	*X*	*X*	*X*	*X*	*X*	*X*	*X*	*X*	*X*

LAMA, long-acting muscarinic antagonist.

Every electronic mail to the participants will include a direct hyperlink to a FAQ on https://coptrin.dk/, and an e-mail that they can contact in case of further questions.

### Sample size

In Denmark, approximately 400,000 people are assumed to have COPD; however, approximately a quarter of them have not been diagnosed yet. We have data showing that 178,000 citizens have collected LAMA medication within the last 6 months. At the same time, we know that every year, there are approximately 20,000 admissions with AECOPD (severe AECOPD) and 6,000 deaths in patients with COPD. Assuming 50% of the AECOPD admissions are either unique or preceding a death, there are 16,000 annual events corresponding to our primary outcome. Thus, the annual risk of a primary outcome is approx. 16,000/178,000 = 8.9%. We hypothesize that evening LAMA administration will reduce the relative risk of the primary outcome by 25%, corresponding to a 2.2% absolute risk reduction. Assuming 84.4% uniformly distributed adherence to the intervention (= approximately 1 in 6 does not adhere), this corresponds to a relative risk reduction of 21.3% = 1.9% absolute risk reduction (average effect among all) in the ITT population. Alpha and power (1-beta) are set at 0.05 (two-sided) and 0.8, respectively. This leads to a needed sample size of 6,662 (3,331 + 3,331).

### Recruitment

Through the Danish Health Data Authority, COP:TRIN will obtain personal identification numbers for all Danish citizens currently treated with LAMAs (all combinations). Recruitment letters will be sent through the official Danish electronic mailbox system (Digital Post). The letter will contain a link to a REDCap questionnaire for the participants to complete if they are interested. The questionnaire will obtain the participant’s personal identification number, select sociodemographic and lifestyle-related variables, confirm current treatment with LAMAs, and obtain consent to participate.

## Assignment of interventions: allocation

### Sequence generation

Patients will be randomized 1:1 to one of the two treatment strategies by computer-generated allocation to either LAMA administration in the morning or evening.

### Concealment mechanism

The concealment mechanism for investigators will be that the database manager and outcome assessors will be blinded. The allocated group will not be concealed from participants.

### Implementation

After providing informed consent, the participant will automatically be provided with a randomization allocation, to either LAMA administration in the evening or the morning, on their screen and sent to their official electronic mailbox, as will the participant be continuously reminded (Table 4).

## Assignment of interventions: blinding

### Who will be blinded

The trial is open-label. Trial participants will not be blinded. The database manager and the outcome assessors will be blinded. Apart from this, all endpoints are assessed using prespecified registry-based definitions to ensure maximal reduction of the risk of bias.

### Procedure for unblinding if needed

N/A, the patient is not blinded to the allocated group.

## Data collection and management

### Plans for the assessment and collection of outcomes

In addition to what is obtained through the initial questionnaire, all information on baseline characteristics will be collected from Danish nationwide registries. This will be supplemented with data from the initial questionnaire on body mass index, smoking status (including pack-years), FEV1, and asthma. Information on adherence and symptom scores for exploratory outcomes will be obtained through questionnaires. The data from nationwide registries include age, gender, exacerbation history, comorbidities, and relevant medication details. All data used to assess clinical endpoints during the study will be collected from Danish nationwide registries.

### Plans to promote participant retention and complete follow-up

The trial will aim to ensure adherence and compliance with the allocated randomized group through electronic reminders (Table 4).

### Data management

All data will be saved on REDCap. A statistician will be responsible for the validity of analyses.

### Confidentiality

Personal information about potential and enrolled participants will be collected, shared, and maintained according to patient–physician confidentiality and GDPR.

### Plans for collection, laboratory evaluation, and storage of biological specimens for genetic or molecular analysis in this trial/future use

N/A

No biological specimens will be obtained in this trial.

## Statistical methods

### Statistical methods for primary and secondary outcomes

The primary and all secondary outcomes will be analyzed using the intention-to-treat (ITT) approach, which encompasses the entire cohort of randomized participants in the study. Additionally, per-protocol analyses will be conducted for patients taking inhalation medication 6–7 days per week at the allocated administration time for the full duration of the study, along with modified ITT (mITT) analyses for those who have taken less than 6 days or more than 2 days per week for any amount of time. These analyses will be performed on both the primary and all secondary outcomes, providing a comprehensive evaluation of the study’s results.

The primary outcome (hospitalization-requiring AECOPD or death from all causes within 12) will be presented as a fraction with percentage and relative risk with a 95% confidence interval. The groups will be compared using a two-sided binomial test. The same principles will be applied to secondary binomial outcomes. For the SABA pick-up rate, the incidence rate and ratios will be calculated and presented (including 95% confidence intervals) and compared using the chi-squared test. Changes in CAT and MRC (respectively) will be reported as median differences with IQR and compared within and between groups with a paired two-sided Wilcoxon signed-rank test and an unpaired two-sided Wilcoxon rank-sum test. A subgroup analysis will be performed on the primary outcome with the following subgroups: adherence group (high adherence: ≥4–5 days per week of adherence), age (≥65 years), asthma overlap, coexisting heart failure, body mass index (>30 kg/), frequent exacerbator (>1 severe exacerbation within 12 months prior to baseline), and treatment with systemic beta-2 antagonist at baseline. A *p*-value of <0.05 will be considered significant.

### Interim analyses and stopping guidance

A Data and Safety Monitoring Board (DSMB) will be appointed and act according to a charter agreed by the investigators and approved by the sponsor. Six months after the recruitment of the last patient, an interim analysis, based on the ITT population, will be performed to evaluate the continuation of the trial. As main statistical measures, an O’Brien-Fleming plot of the primary endpoint and mortality (calculating *Z*-scores) will be performed. The interim analysis will be performed and presented by a sub-investigator not otherwise involved in the data collection or analyses, overlooked by the trial statistician. The data will be presented in a blinded fashion.

At the interim analysis, the event rate and effect size for the primary endpoint, as well as the safety and progress of the study, will be assessed by the DSMB, and follow-up duration may be adjusted accordingly. Safety will be assessed by comparing event rates to the event rate presumption used for the initial sample size determination. The study may stop for futility if it is deemed improbable to achieve a follow-up duration sufficient to detect a clinically important effect size. The study will not stop for superior efficacy at interim analysis. We wish to prioritize increasing the likelihood of having sufficient power to conclude on the secondary endpoints at the final analysis.

### Methods for additional analyses (e.g., subgroup analyses)

Continuous baseline variables will be summarized using means, 95% confidence intervals, medians, and interquartile ranges according to distribution. Categorical variables will be compared using frequencies and proportions. All statistical tests will be two-sided, and *p*-values of <0.05 will be considered statistically significant.

### Methods in analysis to handle protocol non-adherence and any statistical methods to handle missing data

Secondary analyses will include mITT and per-protocol analyses to provide a comprehensive evaluation of the study outcomes. No data on the primary outcome will be missing as national registries will be used for outcome assessment. Only self-reported outcomes such as MRC and CAT can be expected to be missing. It will be assumed all missing data will be missing at random or completely at random to allow for multiple imputations. No imputed data will be presented in the baseline table.

### Plans to give access to the full protocol, participant-level data, and statistical code

The study protocol has been published on www.coptrin.dk.

Participant-level data and statistical code will not be published.

## Oversight and monitoring

### Composition of the coordinating center and trial steering committee

The coordination center is composed of Pradeesh Sivapalan, Valdemar Rømer, Niklas Dyrby Johansen, Tor Biering-Sørensen, and Jens-Ulrik Stæhr Jensen. The trial steering committee is made up of the COP:TRIN organization and has given input to the design of the study. Information on the study has been published by the coordination center on www.coptrin.dk.

### Composition of the data monitoring committee, its role, and reporting structure

The DSMB comprises three independent members who will assess the interim analysis 6 months after the recruitment of the last patient.

### Adverse event reporting and harms

N/A as the trial does not alter the type of medication or dosage of medication already being used.

### Frequency and plans for auditing trial conduct

There will be an independent data safety monitoring committee, as mentioned in 21a. Otherwise N/A.

### Plans for communicating important protocol amendments to relevant parties (e.g., trial participants and ethical committees)

If any changes are made, all relevant parties will be informed promptly by mail and through Trial Setting Committee meetings.

### Dissemination plans

Both negative, positive, and inconclusive results will be submitted for publication in peer-reviewed journals and presented at international scientific conferences. Authorship criteria will be based on Recommendations for the Conduct, Reporting, Editing, and Publication of Scholarly Work in Medical Journals by The International Committee of Medical Journal Editors.

Participant-level data and statistical code will not be published.

## Discussion

Morning symptoms are common in COPD and have an important impact on patient quality of life (QoL) (7). Nighttime administration may lead to better treatment effects in the morning and thus reduce these symptoms among patients.

In addition to morning symptoms, there is evidence that up to 66% of COPD patients have airway obstruction during their sleep leading to desaturation, poor sleep quality, cardiovascular disease, and more frequent exacerbations (8, 9). A randomized trial with 80 patients showed that tiotropium improved oxygen saturation during sleep compared to placebo (10). The average oxygen saturation during sleep was 3% points higher with nighttime administration and 2% points higher with daytime administration compared to placebo. The difference between the two dosing regimens was not statistically significant; however, only 40 patients were included in this comparison, so it may have been insufficiently powered. Furthermore, this study was not able to examine clinical outcomes such as exacerbations or mortality.

Hospitalization-requiring AECOPD has a significant clinical and socioeconomic impact. A large database review of over 73,000 patients with up to 17-year follow-ups found that fewer than half of patients hospitalized for an exacerbation were still alive after 5 years (11). Patients who survive hospitalization-requiring AECOPD are likely to experience significantly impaired QoL and are at increased risk of further exacerbations. The contribution of non-hospitalized exacerbations to mortality rates (i.e., unreported exacerbations that should have warranted hospital treatment) has not been well-quantified (12). Therefore, COPD treatment to prevent hospitalization-requiring AECOPD or death from all causes is a crucial therapeutic goal for patients with COPD. We will aim to reduce exacerbations and mortality with our simple intervention which can have a great impact on both patients and society, including resource consumption during hospitalizations for this large group of patients.

## Ethics statement

The requirement for ethical approval was waived by the Committees on Health Research Ethics for the Capital Region, due to the nature of the study and the absence of alterations in medication or dosage. The studies were conducted in accordance with local legislation and institutional requirements.

## Author contributions

PS: Writing – review & editing, Writing – original draft. VR: Writing – review & editing, Writing – original draft. TW: Writing – original draft. ND: Writing – original draft. MP: Writing – original draft. DM: Writing – original draft. AM: Writing – original draft. JV: Writing – original draft. JE: Writing – original draft. AJ: Writing – original draft. JH: Writing – original draft. TB-S: Writing – original draft. J-UJ: Writing – review & editing, Writing – original draft.
